# Support for phosphoinositol 3 kinase and mTOR inhibitors as treatment for lupus using in-silico drug-repurposing analysis

**DOI:** 10.1186/s13075-017-1263-7

**Published:** 2017-03-11

**Authors:** Daniel Toro-Domínguez, Pedro Carmona-Sáez, Marta E. Alarcón-Riquelme

**Affiliations:** 1 Area of Medical Genomics, Pfizer–University of Granada–Andalusian Government Centre for Genomics and Oncological Research (GENYO), Avda. de la Ilustración 114, PTS-18016 Granada, Spain; 2 Bioinformatics Unit, Pfizer–University of Granada–Andalusian Government Centre of Genomics and Oncological Research (GENYO), Avda. de la Ilustración 114, PTS-18016 Granada, Spain; 3 Unit of Chronic Inflammatory Diseases, Institute of Environmental Medicine, Karolinska Institute, Stockholm, 17177 Sweden

**Keywords:** Systemic lupus erythematosus, Drug repurposing, Lincscloud, Gene expression, Autoimmunity, Drug discovery

## Abstract

**Background:**

Systemic lupus erythematosus (SLE) is an autoimmune disease with few treatment options. Current therapies are not fully effective and show highly variable responses. In this regard, large efforts have focused on developing more effective therapeutic strategies. Drug repurposing based on the comparison of gene expression signatures is an effective technique for the identification of new therapeutic approaches. Here we present a drug-repurposing exploratory analysis using gene expression signatures from SLE patients to discover potential new drug candidates and target genes.

**Methods:**

We collected a compendium of gene expression signatures comprising peripheral blood cells and different separate blood cell types from SLE patients. The Lincscloud database was mined to link SLE signatures with drugs, gene knock-down, and knock-in expression signatures. The derived dataset was analyzed in order to identify compounds, genes, and pathways that were significantly correlated with SLE gene expression signatures.

**Results:**

We obtained a list of drugs that showed an inverse correlation with SLE gene expression signatures as well as a set of potential target genes and their associated biological pathways. The list includes drugs never or little studied in the context of SLE treatment, as well as recently studied compounds.

**Conclusion:**

Our exploratory analysis provides evidence that phosphoinositol 3 kinase and mammalian target of rapamycin (mTOR) inhibitors could be potential therapeutic options in SLE worth further future testing.

**Electronic supplementary material:**

The online version of this article (doi:10.1186/s13075-017-1263-7) contains supplementary material, which is available to authorized users.

## Background

Systemic lupus erythematosus (SLE) is an autoimmune disorder in which the immune system produces autoantibodies against its own cells and tissues leading to chronic inflammation and organ damage. Although some biological pathways are well known to be altered in lupus, such as the type I interferon (IFN) pathway [1], the biological mechanisms behind disease development are poorly understood in general and it has been proposed that genetic and environmental factors are involved [2]. There are many classes of drugs commonly used for SLE treatment, such as corticosteroids, immunosuppressants, nonsteroidal anti-inflammatory drugs, or specific monoclonal antibodies directed against cell surface receptors or cytokines [3]. Nevertheless, the multifactorial nature and the undefined etiology of this disease contribute to the absence of efficient treatments [4].

In the last decade, the widespread use of high-throughput technologies such as gene expression microarrays has enabled access to large collections of gene expression databases that can be exploited for a wide range of applications. In this context, in-silico drug-repurposing analysis based on gene expression data allows us to identify new therapeutic applications for drugs used in other contexts. This technique compares the disease gene expression signature against a large collection of profiles derived from different compounds, measuring the degree of similarity among them. A positive similarity score means that the compound produces a similar gene expression pattern to that of the disease. In the same way, a negative similarity score represents the opposite; that is, the overexpressed genes in the disease appear underexpressed in the drug signature and vice versa. This evidences that the effect of the drug on transcription is opposite to the effect of the disease, and it is reasonable to hypothesize that the drug might be able to reverse the disease gene expression program and the phenotype itself [5].

The Connectivity Map [6] was a pioneer tool that implemented this approach. Since its publication, many studies have proven the potential of this type of analysis to discover new treatments for different diseases such as several types of cancer, muscle atrophy, or inflammatory bowel disease, among others [7].

In this context, Lincscloud [8] has been deployed recently as the successor to the Connectivity Map. This database contains genetic profiles derived from a larger number of drugs and also includes knock-down and knock-in gene experiments, where whole gene expression profiles are measured after inhibiting or overexpressing a single gene. During the last few years there has been an increasing interest in the application of this approach for drug repurposing or target predictions. For example, Johannessen et al. [9] explored the transcriptional connections between cAMP signaling and GPCR pathway-associated drug resistance candidates. Santagata et al. [10] revealed a strong connection between the *HSF1* gene and compounds that inhibit protein translation, while Siavelis et al. [11] proposed new treatments for Alzheimer’s disease.

In this work we performed a drug-repurposing analysis using a collection of gene expression signatures derived from previously published studies of SLE patients and gene expression signatures derived from Lincscloud. This analysis allowed us to establish a set of drug candidates that reverse the SLE signatures and a set of genetic targets, as well as new pharmacological paths in SLE.

## Methods

### Processing gene expression data

We mined the National Center for Biotechnology Information (NCBI) Gene Expression Omnibus (GEO) database [12] to retrieve gene expression datasets from SLE patients. We selected experiments performed in any blood tissue, with case and healthy samples, without any treatment applied in the case of in-vitro samples, and each experiment with more than four replicates. To purposely obtain a heterogeneous dataset we searched for gene expression data from adult and juvenile SLE performed in different microarray platforms. By doing this we considered the patterns conserved across all SLE cases removing differences between SLE clinical types or microarray platform-dependent biases.

Each gene expression dataset was downloaded and processed independently using the R statistical environment. Genes with a high percentage of missing values (more than 15% across samples) were filtered out and remaining missing values were imputed using the average expression values within each group (case or control) of each dataset. We annotated probes to gene symbol identifiers, data were transformed to a logarithm scale, and the median expression value was computed for probes corresponding to the same gene. Differential expression analysis was performed between controls and cases for each dataset using the limma R package. Next we discarded genes presenting *p* > 0.05, and the top 500 most overexpressed and underexpressed genes were selected as the SLE genetic signature from each dataset to be used for further analysis.

### Drug-repurposing analysis

For each independent SLE signature we performed a query on the Lincscloud database and retrieved the list of drugs and knock-in and knock-down genes with high similarity scores. We used as a similarity score the “best score 4” value, which is the proposed threshold in Lincscloud and is calculated as the mean connectivity score across the four cell lines in which the drug or perturbagen connected most strongly to the query.

**Fig. 1:**
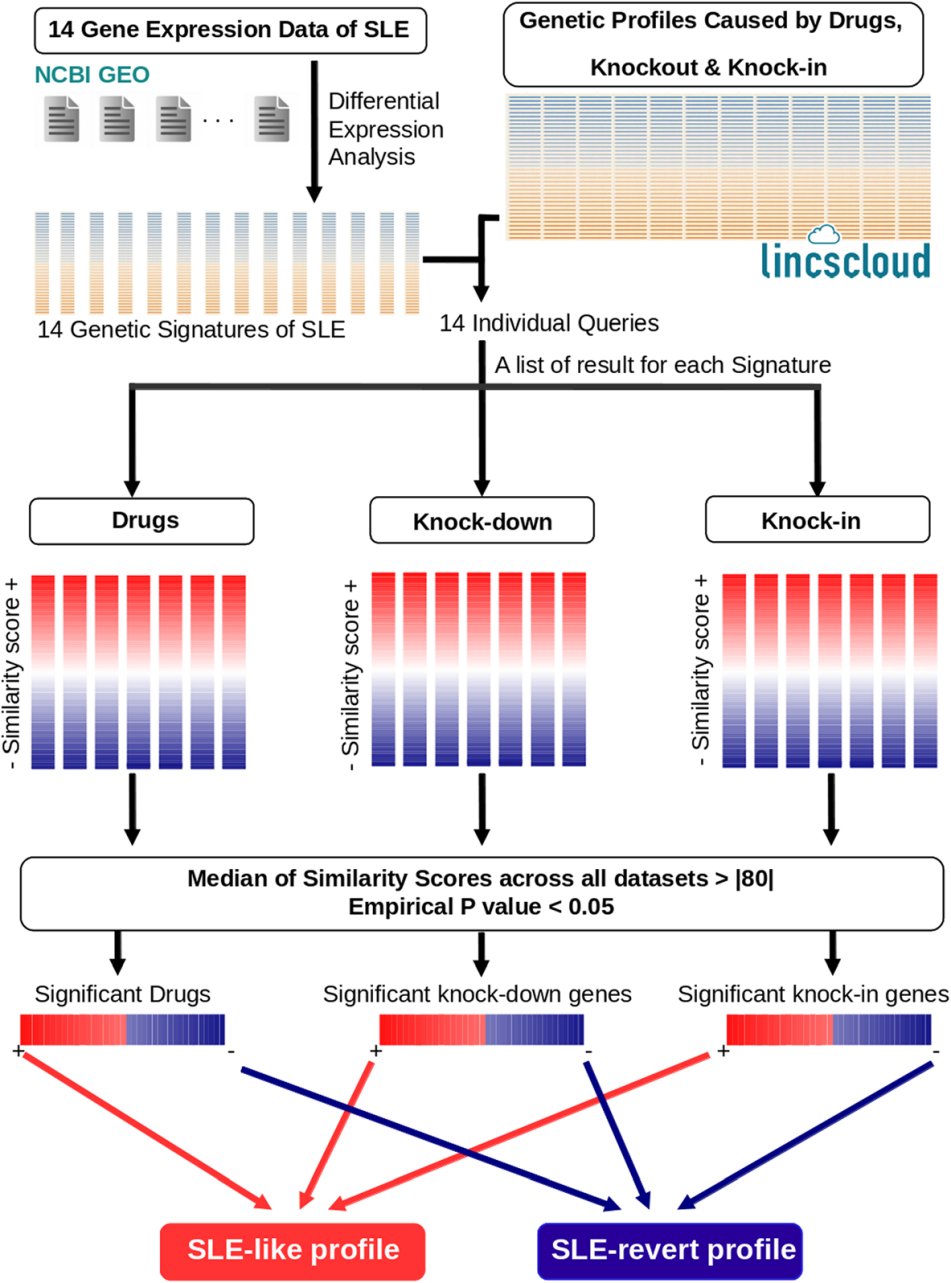
Integrative drug-repurposing analysis. Fourteen signatures of SLE were obtained from 14 different datasets. Each signature was queried on the Lincscloud database and a set of drugs and knock-down and knock-in genes was obtained with similarity scores. The median similarity score and empirical *p* values were calculated to select significant results across all datasets. *Bottom*: summary interpretation of the positively and negatively correlated results. *NCBI GEO* National Center for Biotechnology Information Gene Expression Omnibus, *SLE* systemic lupus erythematosus

To integrate the results from each independent SLE signature, a unique dataset was created where rows represent drugs and columns represent SLE signatures, and each entry of the matrix is the similarity score (best score 4 values) between drugs and SLE signatures. For each drug (row) we calculated the median similarity score across all SLE signatures. To evaluate whether equal or better scores could be obtained by chance, an empirical *p* value was calculated generating 10,000 random datasets permuting rows and columns in the original set of data. We then computed the *p* value as the fraction of permutations having a similarity score equal to or higher than (in absolute value) the observed score. Significant drugs were then selected if they presented *p* < 0.05 and showed a median similarity score > 80. The same procedure was applied to knock-in and knock-down gene expression signatures (see Fig. 1). The results obtained are therefore independent of the cell lines’ inherent gene expression patterns but are consistent with the patterns that are common to all of the SLE signatures.

### Drug-target enrichment analysis

To evaluate whether some drug targets were significantly enriched in the list of obtained drugs we downloaded drug-target information from DrugBank [13], ChEBI [14], and Therapeutic Target Database [15]. Data files from these three databases were parsed and an annotation file was created with information for 131,162 drugs (including synonymous names) and their biological targets. With this information, we associated target genes to the list of drugs in Lincscloud and our list of significant drugs. For drugs without target information in these resources we carefully revised the information available from compound manufacturer catalogs and the associated literature. Drugs without any information in the literature or in databases were discarded from the drug-target analysis.

Fisher’s exact test was applied to evaluate what target genes were statistically overrepresented in the list of significant drugs with respect to the total set of annotated drugs.

## Results

### Analysis of gene expression signatures

After careful exploration we found 10 datasets of SLE in the NCBI GEO, two of which contained samples from juvenile SLE patients. Some of the datasets contained samples from different tissues, which we treated as independent datasets in our analysis. Thus, we identified 14 different tissue-specific datasets that passed the initial filters (see Additional file 1: Sheets 1 and 3). These datasets comprised a total of 327 SLE samples and 173 healthy controls. Each dataset was subjected to quality control and processed as described in Methods, generating 14 individual signatures including different blood tissues (see Additional file 1: Sheet 2).

### Connections between SLE and drug gene expression signatures

**Fig. 2:**
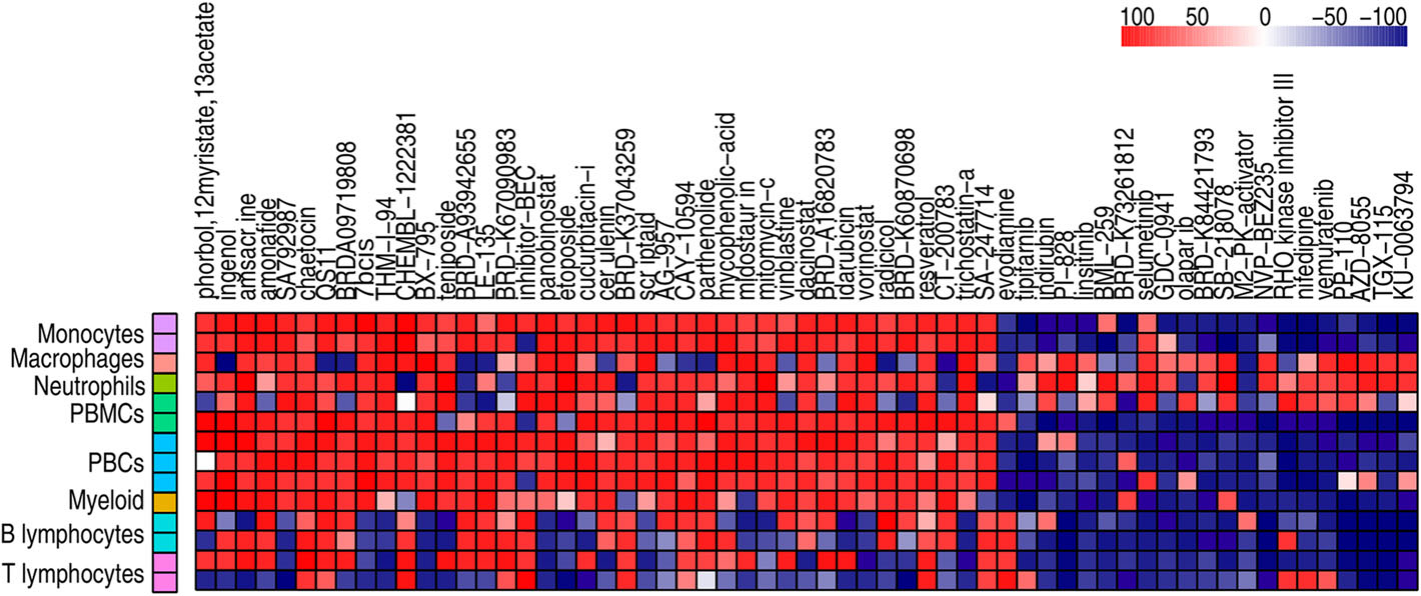
Heatmap of significant drugs representing similarity scores for each drug in the results of each dataset. *Rows*: results of the different datasets used for the analysis. Datasets classified according to the blood cell type (see key). *Columns*: different drugs sorted decreasingly by the median of similarity scores, from left to right (Color figure online)

Our analysis yielded 61 drugs that were significantly associated with the SLE signatures, 40 with similar gene expression patterns and 21 with opposite patterns (see Fig. 2 and Additional file 1: Sheet 4). Some of these compounds have been associated previously with SLE but some others have not been described in this context and hence could be new potential drug candidates (see Discussion). We used the information from DrugBank, ChEBI, and Therapeutic Target Database to annotate target genes for each drug and classify these compounds into groups with the same target.

The analysis of targets common across the list of drugs yielded three sets with similar gene expression signatures that showed significant *p* values, including topoisomerase II inhibitors, histone deacetylase (HDAC) inhibitors, and PKC activators, as well as three groups with negative scores, where we found phosphoinositol 3 kinase (PI3K) inhibitors, cyclin-dependent kinase (CDK) inhibitors, and mammalian target of rapamycin (mTOR) inhibitors (see Table 1). Five different compounds were PI3K inhibitors, providing the most significant *p* value in the enrichment analysis.

**Table 1:** Drugs obtained and their biological targets

Score^a^	Biological target	Action	Drugs	*p* value
+	Topoisomerase II	Inhibitor	Amsacrine, amonafide, teniposide, etoposide, idarubicin	2.829 × 10^–4^
+	HDAC	Inhibitor	Panobinostat, scriptaid, dacinostat, vorinostat, trichostatin A	1.451 × 10^–4^
+	Protein kinase C delta	Activator	Phorbol-12-myristate-13-acetate, ingenol	3.172 × 10^–3^
+	Histone lysine methyltransferase	Inhibitor	Chaetocin	
+	ARFGAP1	Inhibitor	QS11	
+	PDK1	Inhibitor	BX795	
+	Retinoic acid receptor beta	Inhibitor	Le135	
+	Arginase	Inhibitor	Inhibitor Bec	
+	JAK2/STAT3	Inhibitor	Cucurbitacin I	
+	Fatty acid synthetase	Inhibitor	Cerulenin	
+	Src, Bcr-Abl tyrosine kinase	Inhibitor	AG957	
+	PLD2	Inhibitor	CAY10594	
+	IKKβ	Inhibitor	Parthenolide	
+	IMPDH1	Inhibitor	Mycophenolic acid	
+	FTL3	Inhibitor	Midostaurin	
+	DNA	Crosslinker	Mitomycin C	
+	Tubulin	Inhibitor	Vinblastine	
+	Hsp90	Inhibitor	Radicol	
+	Multiple targets	Inhibitor	Resveratrol	
–	PI3K	Inhibitor	PI828, GDC0941, NVP-BEZ235,PP110, TGX115	4.915 × 10^–6^
–	mTOR	Inhibitor	NVP-BEZ235, AZD8055, TGX115, Ku0063794	1.792 × 10^–5^
–	CDK	Inhibitor	BML259, indirubin	1.463 × 10^–2^
–	IKBalfa	Inhibitor	Evodiamine	
–	Farnesyltransferase	Inhibitor	Tipifarnib	
–	IGF1R	Inhibitor	Linsitinib	
–	MAP2K1	Inhibitor	Selumetinib	
–	CHK1	Inhibitor	SB218078	
–	Piruvate kinase	Inhibitor	M2PK activator	
–	Rho kinase	Inhibitor	Rho kinase inhibitor III	
–	Voltage-dependent calcium channel	Inhibitor	Nifedipine	
–	Braf	Inhibitor	Vemurafenib	

Table presents significant drugs with their biological target and their mechanism of action. *p* value calculated for groups of drugs with the same target using Fisher’s exact test

*CDK* cyclin-dependent kinase, *HDAC* histone deacetylase, *mTOR* mammalian target of rapamycin, *PI3K* phosphoinositol 3 kinase

^a^ *+* drugs with positive similarity score, *–* drugs with negative similarity score in regard to SLE signatures

To further explore this result, we used information from the KEGG database [16] to construct a network of the PI3K signaling pathway (see Additional file 2). Interestingly, we found that most of the other drug targets, such as IGF, Rho, mTOR, or CDK, were also playing important roles in the PI3K signaling pathway. PI3K regulates important processes such as cell survival, immune proliferation, anti-apoptotic pathways of immune cells, and immune response linked to interferon signaling and cytokine signaling pathways [17], all important and impaired in SLE. We also obtained dual inhibitors of PI3K and mTOR such as NVP-BEZ235 [18]. Other drug targets of the PI3K signaling pathway have been related with SLE or other SLE-like disorders, such as CDK inhibitors, recently proposed to be used for treatment of some autoimmune disorders [19], or inhibitors of the mitogen-activated protein kinase (MAPK) signaling pathway [20].

### Study of gene effect-caused profiles

We obtained seven knock-in and 90 knock-down genes with a positive similarity score that produce an SLE-like profile, and 50 knock-down genes with a negative similarity score (see Table 2 and Additional file 1: Sheet 4) that reverse the SLE profile (genes up-regulated in the disease signature are down-regulated in the drug signature, and vice versa). Many genes have been already described in SLE, such as *CD40* [21], interferon-related genes, and translation initiation factors, such as *EIEF4* [22, 23]. Additional functional analyses with these genes are described in Additional file 3. Interestingly and in agreement with our previous analysis, we found that the gene expression signature associated with knock-down genes such as *PI3K* or *IGF1R* show a negative similarity score. That is, the inhibition of these genes could reverse the gene expression profile induced by SLE. This is consistent with the fact that gene expression profiles of drugs which inhibit these genes showed a negative score with respect to the SLE signatures.

**Table 2:** Significant knock-down and knock-in genes obtained

Score	Type of experiment	Genes
+	Knock-in	*IFNB1*, *IFNG*, *CD40*, *BCL10*, *KLF6*, *LYN*, *TIRAP*
+	Knock-down	*CLCN3*, *PPP1R14B*, *LMNB2*, *TBX2*, *PMM2*, *MYC*, *ATP6V1F*, *MAX*, *PEPD*, *PUF60*, *PHB2*, *AKR1A1*, *BTG1*, *ABHD2*, *TFDP1*, *PAX8*, *FOSL2*, *NT5E*, *RRM1*, *NR2F6*, *RAMP1*, *RYK*, *CISH*, *PPP2R1A*, *CD14*, *UFD1L*, *HTRA1*, *SLC35A1*, *TWF2*, *NNT*, *HOMER2*, *HS2ST1*, *ZNF768*, *GGT1*, *DFFB*, *HSPA2*, *PRKDC*, *ARPC5*, *NFKBIA*, *SLC39A8*, *THAP11*, *GSTP1*, *ETV1*, *GCAT*, *KIAA0907*, *DLX3*, *ELK1*, *PIAS4*, *MEOX2*, *GPER*, *NRAS*, *TCEB3C*, *KIF2C*, *POLR2F*, *CTBP2*, *CHAF1B*, *CEP55*, *HOOK2*, *ZNF8*, *NDUFB7*, *NISCH*, *HOXC10*, *AQP12A*, *YES1*, *PSMD5*, *JAG1*, *MDH2*, *POLR2I*, *DDF1*, *HRAS*, *HDAC10*, *SLC25A14*, *MED7*, *HMGCR*, *PDXP*, *FDX1*, *NIPBL*, *PRKAG3*, *PPIA*, *EIF2AK3*, *B4GALT1*, *UCK2*, *JUN*, *MED4*, *YBX1*, *BUB1B*, *CRCP*, *MED1*, *HDAC11*, *SBNO1*
–	Knock-down	*MITF*, *ETFA*, *PIP4K2B*, *VRK2*, *SPEN*, *NSDHL*, *ZNF586*, *GNPDA1*, *SIX4*, *PARN*, *DUSP14*, *IQGAP1*, *LRRK2*, *GPR123*, *SF1*, *FEZ2*, *IPMK*, *SAT1*, *ELF4*, *RPTOR*, *EIF4E*, *ARL3*, *KARS*, *CSNK1A1*, *SPTLC2*, *MEN1*, *SNX17*, *VEGFC*, *PPP3CA*, *BNIP3*, *ERBB3*, *ERO1L*, *COPB2*, *SERPINC1*, *AK4*, *HLA_A*, *PIK3CA*, *PIK3C2A*, *IGF2R*, *LYPLA1*, *STX4*, *ATM*, *ESPL1*, *IGF1R*, *ST3GAL5*, *MTOR*, *GRN*, *HSP90AA1*, *PRPF4B*, *TM9SF3*

Table presents knock-in genes with positive similarity score (score +), and knock-down genes with positive and negative similarity score (score –). The genes are sorted into each list by median of similarity scores across all dataset. No knock-in signatures were found with significant negative similarity score

## Discussion

In this study we performed a systematic screening for drugs or genes that induced similar or opposite gene expression programs to signatures from SLE patients. We integrated signatures from different blood cell populations and SLE subtypes in order to identify consistent and conserved profiles, reducing considerably the false positive ratio. In this analysis, we found 40 drugs (see Additional file 1: Sheet 4) with a positive similarity score, which induces changes similar to the SLE phenotype. In this set of compounds, HDAC, topoisomerase II, and PKC were the more significant targets. Many of these drug targets are key factors in biological processes that are altered in SLE. For example, HDAC inhibitors have been related to impairment of immune processes described in lupus, such as autophagy [24], although there is contradictory information about the role of some isoforms of HDAC in the immune system [25, 26]. A recent study shows that HDAC inhibitors may be suitable for treatment of autoimmunity, while primary responses to the same inhibitors were greatly impaired, probably explaining the contradiction between the positive similarity score we obtained and the potential use of HDAC inhibitors in SLE [27]. In addition, Lohman et al. [28] showed that HDAC inhibitors have anti-inflammatory activity which is inversely correlated with dose, amplifying the production of inflammatory mediators at concentration > 3 μM. In another context, treatment of human cells with topoisomerase II inhibitors such as etoposide has been shown to induce interferon-stimulated genes [29].

Other positively correlated compounds are phorbol-12-myristate-13-acetate and ingenol, the former of which has been used to stimulate the immune response and the interferon signaling pathway [30]. These drugs are protein kinase C (PKC) activators, a protein with some isoforms associated with SLE. In this context, the use of PKC inhibitors has been proposed as treatment for autoimmune disorders [31, 32] due to their induced increase in proliferation of regulatory T cells (Tregs). In addition, deficient MEK/ERK signaling pathway is related to SLE and cytokine generation [33] through impaired PKC activation. This pathway has also been proposed as a potential therapeutic target for rheumatoid arthritis [34]. Another compound with a high positive similarity score was LE-135, which is a retinoic acid receptor inhibitor. The use of retinoic acid has been also related to an improvement in SLE recovering the Treg balance [35, 36].

Attending to drugs with negative similarity scores, we identified 21 compounds that induce opposite gene expression programs with respect to SLE signatures (see Additional file 1: Sheet 4). Almost all of them act in the same processes, down-regulating the immune response and the proliferation of immune cells. PI3K was the most significant in the target enrichment analysis, due to a set of PI3K inhibitors. PI3K inhibitors have been reported to ameliorate the effects of SLE and other autoimmune disorders in animal models [37–39]. In addition, mTOR was also found as a significantly enriched target associated with mTOR inhibitors such as NVP-BEZ235, AZD8055, TGX115, or Ku0063794.

Recent experimental evidence suggests that mTOR inhibitors may provide a new therapeutic strategy for the treatment of SLE patients [40]. Indeed, PI3K and mTOR act in the same signaling cascade [38] promoting the interferon and cytokine signaling pathways [41].

Complementarily, the analysis of gene-caused profiles defined a set of genes – both described and not previously described in SLE – that could play an important role in the development of the disease. Some of these were interferon-related genes, transcriptional and translational factors, and a set of biological pathways related to these genes including the PI3K and the insulin signaling pathways, immune response, or transcriptional and translational processes (Additional file 2). These results are highly consistent with the analyzed list of drugs and also support that the inhibition of PI3K signaling could improve the SLE phenotype. The evidence presented here should lead not only to testing of PI3K inhibitors as potential SLE treatment, but also to actively testing any other compound obtained, such as the insulin growth factor receptor inhibitors that crosstalk with the PI3K and mTOR pathways or the Rho kinase inhibitors.

Although the Lincscloud database contains mostly experiments carried out in cancer cell lines, the integration of different SLE signatures and the inclusion of summarized drug signatures from different cell populations enable one to establish global associations based on ubiquitous expression across different cell lines. In-silico analyses are often exploratory studies and should be confirmed by in-vitro or in-vivo experiments. In this sense, previous experiments already provide evidence that PI3K inhibitors ameliorate the SLE phenotype in animal models [37–39], and that of other autoimmune disorders, although these drugs are not used clinically. Our results would therefore provide further support for the inhibition of the PI3K signaling pathway to treat SLE.

## Conclusions

We performed an integrative in-silico drug-repurposing exploratory analysis based on comparing gene expression data of SLE against gene expression profiles produced by perturbagens from the Lincscloud database. Our analysis is designed to reduce the biases of using different microarray platforms and the heterogeneity of SLE, leading to discovery of conserved genetic patterns across different disease states or cell types. We identified a set of pathways related to biological processes impaired in SLE, compounds, and drug targets with potential therapeutic interest for SLE treatment. Based on the results, we highlighted PI3K and mTOR as good candidates and PI3K signaling pathway inhibitors as potential treatment options that are interesting enough to be further explored, although we described other targets that could also be further evaluated to test their effect in improving the phenotype of SLE, such as PKC, MAPK, or other specific kinases. This type of analysis has seldom been performed for autoimmune diseases and can provide novel therapeutic approaches for heterogeneous and multifactorial disorders, such as SLE.

## Additional files

Additional file 1: presents information about quality control of the data, SLE signatures, and significant results obtained: Sheet 1 shows information about datasets including their GEO identifiers, the SLE state, the cell type, and the microarray platform for each, number of control and case samples, PubMed identifier, and date of publication; Sheet 2 shows results of the quality control based on the percentage of missing values and the number of significant genes in each dataset of SLE; Sheet 3 shows the signatures of each dataset used to query on Lincscloud, significant genes sorted by fold change in each signature; and Sheet 4 shows the lists of drugs and knock-in and knockdown genes with similarity scores, median of similarity scores across datasets, and significance values (XLSX 182 kb)
Additional file 2: is a figure showing the PI3K molecular signaling pathway. Plot constructed based on the information of different PI3K interaction graphs from the KEGG database. *Red*, drug targets with positive similarity scores; *blue*, drug targets with negative similarity scores (PDF 373 kb)
Additional file 3: shows a description of the functional analysis of gene targets obtained and their significance, and is divided into three sections: methods, results, and references. (PDF 116 kb)

## Abbreviations

CDK: Cyclin-dependent kinase
HDAC: Histone deacetylase
IFN: Interferon
MAPK: Mitogen-activated protein kinase
mTOR: Mammalian target of Rapamycin
NCBI GEO: National Centre for Biotechnology Information Gene Expression Omnibus database
PI3K: Phosphoinositol 3 kinase
PKC: Protein kinase C
SLE: Systemic lupus erythematosus
Treg: Regulatory T cell
