# Supplementary figures and images for: Support for phosphoinositol 3 kinase and mTOR inhibitors as treatment for lupus using in-silico drug-repurposing analysis
Source: Arthritis Res Ther. 2017 Mar 11;19:54. doi: 10.1186/s13075-017-1263-7 (PMC5346251; doi:10.1186/s13075-017-1263-7)

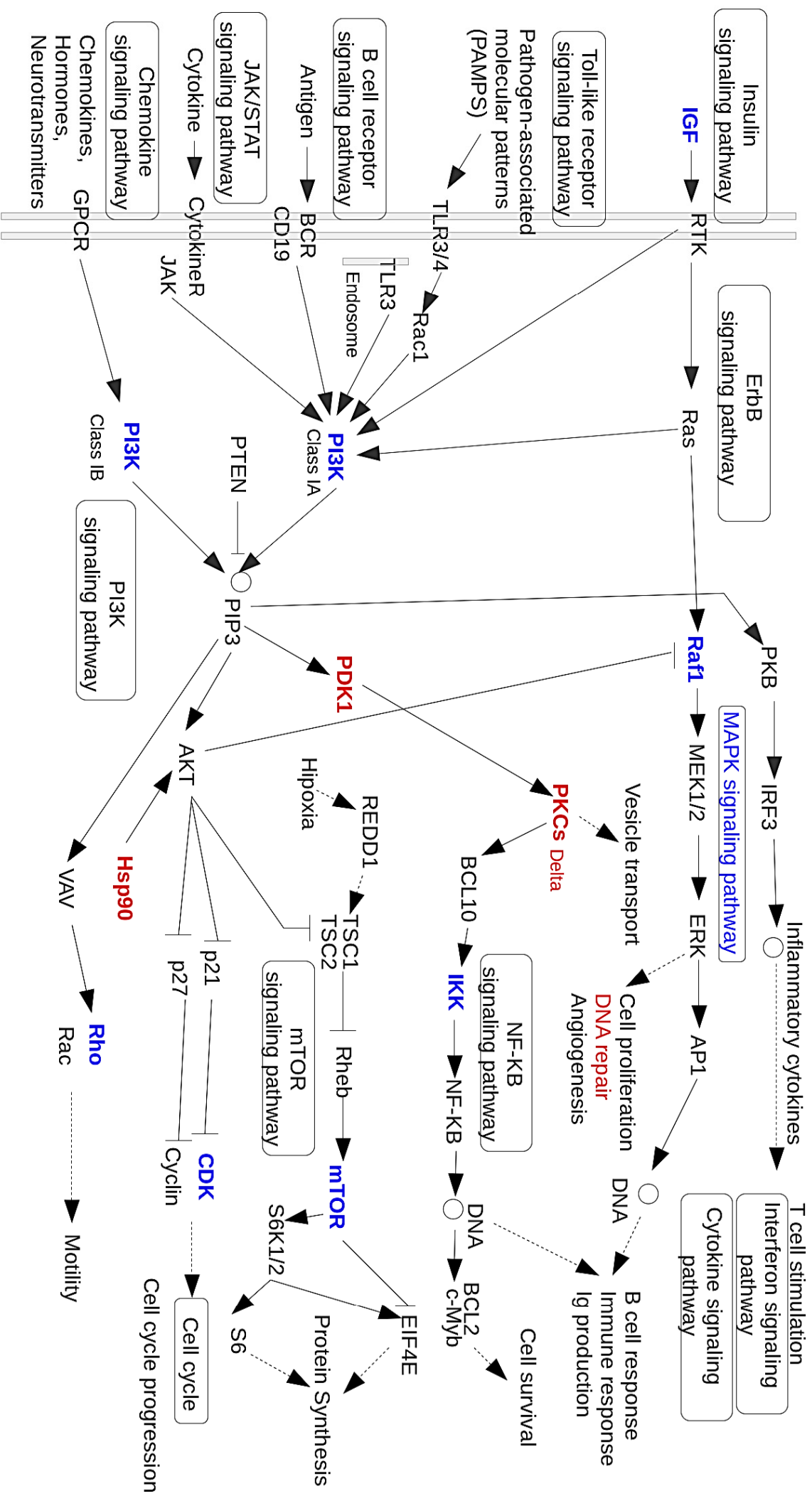

Supplement: Additional file 2: — is a figure showing the PI3K molecular signaling pathway. Plot constructed based on the information of different PI3K interaction graphs from the KEGG database. Red, drug targets with positive similarity scores; blue, drug targets with negative similarity scores (PDF 373 kb) [file 13075_2017_1263_MOESM2_ESM.pdf]
