# Supplementary material for: Support for phosphoinositol 3 kinase and mTOR inhibitors as treatment for lupus using in-silico drug-repurposing analysis
Source: Arthritis Res Ther. 2017 Mar 11;19:54. doi: 10.1186/s13075-017-1263-7 (PMC5346251; doi:10.1186/s13075-017-1263-7)
Supplement: Additional file 3: — shows a description of the functional analysis of gene targets obtained and their significance, and is divided into three sections: methods, results, and references. (PDF 116 kb) [file 13075_2017_1263_MOESM3_ESM.pdf]

Additional File 2: Gene analysis

From the list of knock down and knock-in genes that show high similarity scores to the query signatures we defined those with positive scores as SLE inducer genes and genes with negative similarity scores as therapeutic genes. Similarly, the SLE inducer genes can be therapeutic genes if their expression is reversed. We analyzed the main Gene Ontology biological processes associated to this list of genes with the Enrichr web tool [1] in order to obtain an overview of potentially affected pathways and their relation with impaired pathways in SLE. In addition, we used the Center for Therapeutic Target Validation web tool [2] to evaluate which genes from our results have been previously described in the context of SLE or autoimmunity. This tool establishes relationships between potential drug targets and diseases by evidence based on text mining of research articles, drug-target information from the ChEMBL database, RNA expression, somatic mutations, genetic associations, affected pathways from Reactome database and animal model phenotypes.

Functional analysis of these gene lists yielded a set of pathways related to the immune system from the list of knock-in experiments, while biological pathways such as transcriptional and translational processes and metabolic processes showed a positive similarity score from the list of knock-down experiments (see figure 1A).

We obtained a set of 26 genes related with lupus using the Center for Therapeutic Target Validation (see figure 1B). The 82.99% of all genes have been linked to other autoimmune diseases among which are rheumatoid arthritis, Sjögren's syndrome, multiple sclerosis, diabetes mellitus type I or psoriasis, but not with SLE. So, the genes we obtained are related to autoimmunity processes and could be studied as potential new targets for SLE as well.

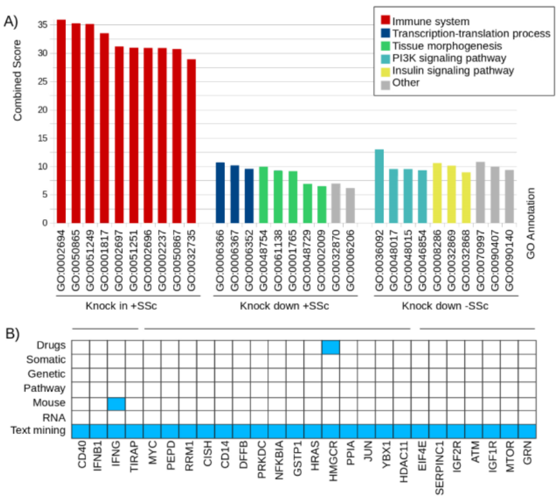

Figure 1. Functional analysis of knock-down and knock-in genes. A) The figure shows the top 10 most related pathways based on the combined score. The x-axis represents significant GO terms obtained and the y-axis the combined score calculated with the Enrichr web tool. The biological processes have been grouped into five more general pathways: Immune system, phosphoinositol signaling pathway, insulin signaling pathway, transcription-translation processes, and morphogenesis. Gene ontology identifiers are provided in additional file 1 (Sheet: 5). B) The plot represents the association (marked in blue) between the knock-down and knock-in genes and SLE. The y-axis contains the type of association based on the Center for Therapeutic Target Validation tool. Genes are in the x-axis. +SSc: positive similarity score; -SSc: negative similarity score.

References

1. Chen EY, Tan CM, Kou Y, Duan Q, Wang Z, Meirelles GV, Clark NR, Ma'ayan A: **Enrichr: interactive and collaborative HTML5 gene list enrichment analysis tool**. *BMC Bioinformatics* 2013, **14**:128.

2. **CTTV** [www.targetvalidation.org]
